# A Novel GnRH-Based Immunocastration Vaccine Modulates Growth, Reproductive and Meat Quality Traits in Male Leizhou Goats

**DOI:** 10.3390/ani16060924

**Published:** 2026-03-15

**Authors:** Mengzhen Luo, Liqin Han, Yueming Yuan, Liuxia Lin, Da Feng, Siyi Hu, Mei Zhou, Namula Zhao, Rui Gao, Shangquan Gan

**Affiliations:** Guangdong Engineering Research Center of Livestock and Poultry Bio-Breeding, College of Coastal Agricultural Sciences, Guangdong Ocean University, Zhanjiang 524088, China; luomengzhen@stu.gdou.edu.cn (M.L.); 15085350116@stu.gdou.edu.cn (L.H.); yuanyueming2@stu.gdou.edu.cn (Y.Y.); linliuxia66@163.com (L.L.); fda_1020@163.com (D.F.); husiyi@stu.gdou.edu.cn (S.H.); 18586026786@163.com (M.Z.); zhaonamula@gdou.edu.cn (N.Z.)

**Keywords:** GnRH vaccine, surgical castration, testicular development, meat quality traits

## Abstract

Castration is widely used in goat production to improve meat quality and manage reproduction, yet conventional surgical methods raise significant animal welfare concerns. Immunocastration targeting gonadotropin-releasing hormone (GnRH) offers a promising, welfare-friendly alternative, but its efficacy in slow-growing indigenous breeds remains poorly defined. In this study, we developed a novel tandem-repeat GnRH(30) recombinant vaccine and evaluated its effects on growth performance, reproductive function, and meat quality in male Leizhou goats. Eighteen six-month-old bucks were assigned to an immunocastration group (IM), surgical castration group (SC), or intact control group (IC). Vaccinated goats produced sustained anti-GnRH antibodies and exhibited significantly suppressed testosterone levels comparable to surgical castrates. Immunocastration induced marked testicular atrophy, disrupted spermatogenesis, reduced semen volume and sperm motility, and increased sperm abnormalities. Importantly, early growth performance and final body weight were not significantly affected. Compared with intact males, both IM and SC goats showed improved meat quality traits, including reduced drip loss and shear force, accompanied by increased intermuscular fat deposition, with overall patterns in the IM group closely resembling those of surgical castration. Overall, these findings indicate that the GnRH(30) vaccine can effectively suppress spermatogenesis and improve meat quality without affecting growth, providing an effective technical approach for castration management in indigenous goats.

## Simple Summary

Castration is commonly used in goat farming to reduce aggressive behavior and improve meat quality, but traditional surgical castration causes pain and raises animal welfare concerns. Immunocastration is a modern alternative that uses vaccination to temporarily block reproductive hormone activity. In this study, we developed a new gonadotropin-releasing hormone (GnRH) vaccine and tested it in male Leizhou goats. The vaccinated goats produced antibodies that reduced testosterone levels and suppressed testicular development and sperm quality and production, similar to surgical castration. Early growth was not affected. Meat quality traits, including tenderness and water-holding capacity, improved and were comparable to surgical castrations. These findings indicate that the GnRH vaccine can effectively replace surgical castration while maintaining growth and meat quality.

## 1. Introduction

Castration is a long-established husbandry practice used to improve meat quality, facilitate management, and control reproduction by suppressing gonadal function [1,2]. Surgical castration, the conventional approach, eliminates sex hormone secretion through testicular removal. This reduces androgen-related meat defects, minimizes aggression and sexual behaviors, and redirects nutrients toward muscle and fat deposition, thereby enhancing meat quality [3,4,5,6]. The technique is widely applied in livestock production [7,8,9,10,11]. However, surgical castration is inherently invasive, causing acute pain, physiological stress, and increased risk of infection, which raises substantial animal welfare and economic concerns [1,12,13,14,15]. These limitations have driven the search for alternative strategies that retain production benefits while reducing welfare costs.

Immunocastration emerged in the late twentieth century as a non-surgical alternative [16,17,18]. Vaccination targeting gonadotropin-releasing hormone (GnRH), the central regulator of the hypothalamic–pituitary–gonadal axis, is currently the most successful strategy [19,20]. By inducing antibodies against endogenous GnRH, immunocastration suppresses gonadotropin release and testosterone synthesis, leading to temporary and potentially reversible reproductive inhibition [21]. Compared with surgical methods, it is minimally invasive and better aligned with modern welfare-oriented livestock systems [21,22]. Its efficacy has been demonstrated in rodent models, where spermatogenesis was profoundly suppressed [23,24], and in commercial livestock such as pigs, cattle, and sheep, where reductions in aggressive behavior and improvements in meat traits have been reported [25,26].

Despite these advances, challenges remain in vaccine design. The GnRH decapeptide is small and poorly immunogenic. Current vaccine strategies typically conjugate GnRH to carrier proteins such as keyhole limpet hemocyanin (KLH) to enhance immune responses [27,28]. Although effective, this approach introduces irrelevant antigenic determinants, potentially reducing the effective concentration of target epitopes and contributing to species-specific variability and the need for repeated immunizations [29].

To overcome these limitations, our research group previously developed a tandem-repeat antigen composed of multiple copies of the mature GnRH decapeptide arranged in series [23,24]. In rodent models, this construct induced high anti-GnRH antibody titers, reduced testosterone to extremely low levels within two weeks after primary immunization, decreased testicular size to approximately 15% of baseline, and suppressed fertility for more than 36 weeks. These findings demonstrated sustained and effective immunocastration in laboratory animals. However, its performance and production relevance in mammalian livestock species have not yet been evaluated.

Based on the above rationale, the present study evaluated the application of a tandem-repeat GnRH(30) vaccine in male Leizhou goats. We investigated its effects on growth performance, reproductive suppression, and meat quality traits to determine its feasibility as a non-surgical alternative to conventional castration in indigenous goat production systems.

## 2. Materials and Methods

### 2.1. Animals and Experimental Design

A total of eighteen healthy 6-month-old male Leizhou goats with similar body weight (10.59 ± 1.07 Kg) were obtained from the Conservation Farm of Guangdong Ocean University. Following a eight-week acclimation period, the animals were randomly assigned to three experimental groups (n = 6 per group): an immunocastration group (IM), a surgical castration group (SC), and an intact control group (IC). All treatments were performed according to established protocols [30]. In the immunocastration group, mild transient swelling occurred at injection sites in some animals but resolved spontaneously, with no severe reactions or behavioral changes. The surgical castration group underwent rubber ring ligation, a method with minimal infection risk; no post-operative complications were observed. The intact control group remained normal throughout. All goats were maintained under standardized management conditions with ad libitum access to feed and water. All animal procedures conducted in this study were approved by the Animal Care and Use Committee of Guangdong Ocean University, College of Animal Science (Approval code: GDOUNXY-068-2024). All experiments were performed in accordance with the relevant guidelines and regulations established by this committee, as well as the national (China) and international standards for the care and use of agricultural animals in research.

**Figure 1:**
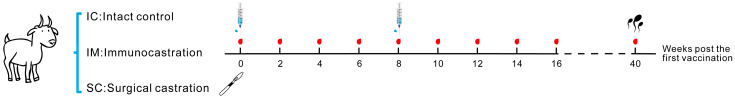
Schematic overview of the experimental design. Note: Animals were randomly assigned to three groups: IC (Intact Control), IM (Immunocastration), and SC (Surgical Castration). In the IM group, animals received the first immunization at week 0 followed by a booster immunization at week 8 (indicated by syringe icons). The SC group underwent surgical castration at the designated time point (indicated by scalpel icon). Blood sampling was conducted at 0, 2, 4, 6, 8, 10, 12, 14, … and 40 weeks post first vaccination (red dots). Semen collection and final evaluation were performed at week 40. The timeline illustrates the longitudinal monitoring schedule following the first immunization.

To minimize potential confounding effects of castration on skeletal development and overall growth performance, the immunization protocol was scheduled to coincide with near completion of gonadal development in Leizhou goats. Specifically, goats in the IM group received a first intramuscular injection of 1.5 mg of the novel GnRH vaccine at 8 months of age (defined as week 0), when the testes were approaching sexual maturity. A booster immunization was administered eight weeks later (approximately 10 months of age) to enhance and sustain the immune response (Figure 1).

Blood samples and body weight measurements were collected at two-week intervals throughout the experimental period, beginning from the first immunization. Testicular dimensions were also measured biweekly starting from the first immunization (week 0). The longitudinal and transverse diameters of both testes were measured using digital calipers. All measurements were performed by the same trained technician to ensure consistency (Figure 1). In accordance with local commercial slaughter practices for Leizhou goats (typically around 18 months of age), all animals were humanely slaughtered eight months after the booster immunization (approximately 18 months of age). At slaughter, tissue samples—including skeletal muscle, testis, and epididymis—were harvested for subsequent histological and molecular analyses.

### 2.2. Expression, Purification, and Vaccine Formulation of the Novel Tandem Recombinant GnRH(30) Protein

The recombinant plasmid pET-28a-GnRH(30) was synthesized by BGI (Shenzhen, China). A DNA fragment encoding the GnRH(30) gene, flanked by *Nco I* and *Xho I* restriction sites, was cloned into the corresponding sites of the pET-28a expression vector. The resulting recombinant plasmid was transformed into *E. coli* BL21 (DE3) competent cells [31]. Positive clones were selected and cultured under optimized conditions to induce protein expression, which involved determining the optimal concentration of isopropyl β-D-1-thiogalactopyranoside (IPTG), temperature, and incubation duration. The recombinant GnRH(30) protein was purified from the bacterial lysates. For vaccine preparation, the purified protein was emulsified with an optimal adjuvant (selected from candidates 206 V, 201 V, and 1313 V) at a predetermined ratio.

### 2.3. Assessment of Growth Performance and Testis Measurements

The body weight of all goats was recorded with a sensitive digital scale at biweekly intervals from the initial immunization (week 0) until the end of the experimental period. Carcass weight and visceral organ weights (heart, liver, spleen, lungs, and kidneys) were measured immediately following slaughter.

Testicular dimensions (length and width) were measured at biweekly intervals throughout the experimental period using a vernier caliper. Testicular length was determined as the distance from the caput epididymis to the cauda epididymis along the longitudinal axis, while testicular width was measured at the widest point perpendicular to the longitudinal axis. To account for scrotal skin thickness, the skinfold thickness (two layers) was measured concurrently and subtracted from the initial length and width values to obtain the actual testicular dimensions, following the method described by Ugwu et al. [32]). The corrected average measurements for both testes were recorded to the nearest 0.01 cm. All measurements were performed by the same trained technician to ensure consistency.

### 2.4. Serum GnRH Antibody and Testosterone Level Assays

Venous blood samples were collected, allowed to clot at 37 °C for 2 h, and centrifuged at 2000× *g* for 10 min to obtain serum, which was stored at −80 °C until analysis.

Serum GnRH antibody levels were determined by an indirect enzyme-linked immunosorbent assay (ELISA). Briefly, 96-well plates were coated with GnRH protein (100 ng/well) and blocked with 2% bovine serum albumin (BSA)(Macklin, Shanghai, China). Test sera (diluted 1:400) were added, followed by incubation with a horseradish peroxidase (HRP)-conjugated rabbit anti-goat IgG secondary antibody (ZSGB-Bio, Beijing, China). The optical density (OD) was measured at 450 nm after color development [23].

Serum testosterone concentrations were quantified using a commercial goat testosterone ELISA kit (mmbio, Yancheng City, China), strictly following the manufacturer’s instructions [23].

### 2.5. Evaluation of Testicular and Epididymal Morphology and Histology

Immediately after slaughter at approximately 18 months of age, testes and epididymides were harvested and weighed. Their longitudinal and transverse diameters were measured. Tissue samples from one side were fixed in 4% paraformaldehyde, embedded in paraffin, sectioned, and subjected to hematoxylin and eosin (H&E) staining. The morphological structure of the seminiferous tubules and epididymal ducts was examined under a light microscope. All fixatives and staining reagents were procured from Servicebio (Wuhan, China).

### 2.6. Analysis of Semen Quality

Semen was collected from all groups via electro-ejaculation and transported to the laboratory at 25 °C within 30 min. Sperm concentration, motility, and movement patterns were analyzed using a computer-assisted sperm analysis (CASA) system (GSA-810 SERIES, HARIOMED, Guangzhou, China). Sperm morphology was assessed using eosin–nigrosin staining smears [24,33].

### 2.7. Determination of Meat Quality Parameters

*Longissimus dorsi* muscles were collected for meat quality analysis. Muscle pH was measured at 45 min and 24 h postmortem using a portable pH meter. Shear force was determined using a texture analyzer (TA-XT2i) equipped with a Warner–Bratzler (SMS, UK) shear blade on cooked meat samples. Drip loss was calculated as the percentage weight loss after suspending a meat sample in an inflated plastic bag at 4 °C for 24 h. Moisture content was measured by the weight difference before and after freeze-drying. Meat color was also evaluated [34].

### 2.8. Statistical Analysis

All data are presented as mean ± standard deviation (SD). Statistical analyses were performed using SPSS software (version 26.0, IBM, USA). For single-time-point measurements (e.g., tissue weights, sperm data), one-way analysis of variance (ANOVA) was applied. For longitudinal data (e.g., body weight, hormone levels), repeated-measures ANOVA was used, with Greenhouse–Geisser correction when sphericity was violated. Post hoc comparisons were conducted using Bonferroni’s test. A *p*-value of less than 0.05 was considered statistically significant.

## 3. Results

### 3.1. Immunogenicity of the Novel GnRH(30) Recombinant Vaccine in Leizhou Goats

**Figure 2:**
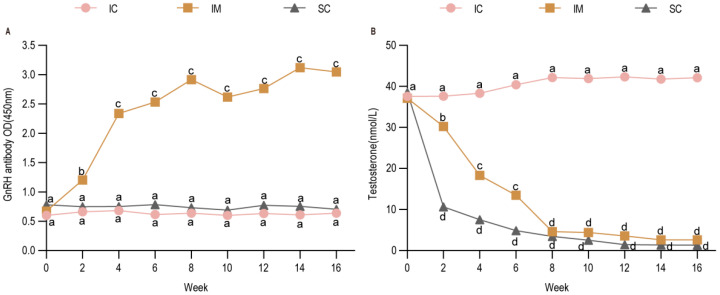
Changes in GnRH Antibody and Testosterone Levels in Three Groups. (**A**) Changes in serum anti-GnRH antibody titers. (**B**) Changes in serum testosterone levels. Note: Two weeks post-immunization, GnRH antibody levels in the IM group were significantly higher than those in the SC and IC groups, while the non-immunized SC and IC groups exhibited low GnRH antibody titers. Testosterone levels in the IC group were significantly higher than those in the SC and IM groups. From 10 weeks post-immunization onward, no statistically significant difference was observed between the SC and IM groups. Statistical analysis was performed using two-way ANOVA. Data are presented as means ± SD. Different lowercase letters (a, b, c, and d) indicate significant differences among groups at each time point: groups sharing the same letter are not significantly different (*p* > 0.05); groups with different adjacent letters (e.g., a vs. b) indicate *p* < 0.05; groups with non-adjacent letters (e.g., a vs. c) indicate *p* < 0.01; groups with a vs. d indicate *p* < 0.001. IC: Intact control group; IM: Immunocastration group; SC: Surgical castration group; OD: optical density.

Following the primary immunization (week 0), serum GnRH antibody levels in the IM group increased rapidly within two weeks and remained at a relatively stable plateau for the subsequent 8 weeks. A booster immunization elicited a further, though modest, increase in antibody titers. In contrast, GnRH antibody levels in both the IC and SC groups were significantly lower than those in the IM group from week 2 onwards (IM vs. IC: *p* < 0.01; IM vs. SC: *p* < 0.01), a significant difference that persisted fifteen weeks (Figure 2A). Concurrently, serum testosterone profiles exhibited an inverse relationship with GnRH antibody levels. Testosterone concentrations in the IM and SC groups were comparable and both were significantly lower than those in the IC group (IC vs. IM: *p* < 0.01; IC vs. SC: *p* < 0.01) (Figure 2B). These results demonstrate that the two-dose regimen of the novel GnRH(30) vaccine successfully induced specific antibody production and effectively suppressed testosterone secretion in Leizhou goats, confirming its good immunogenicity in this breed.

### 3.2. Impact of GnRH(30) Vaccination on Growth Performance

**Figure 3:**
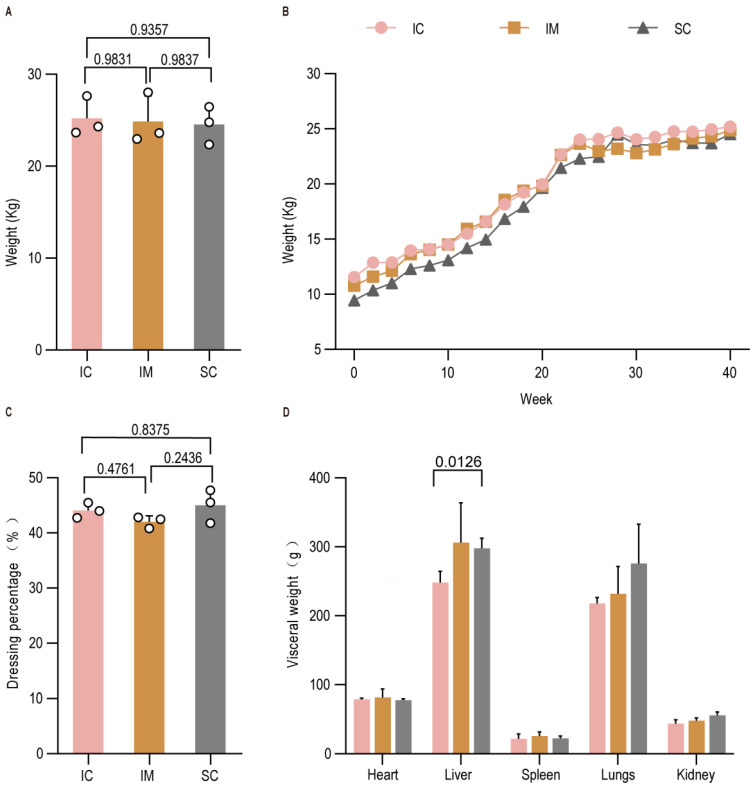
Effects of GnRH immunization on growth performance of Leizhou goats. (**A**) Live weight; (**B**) Dynamic changes in body weight; (**C**) Dressing percentage; (**D**) Visceral organ weight. Data are presented as mean ± SEM.

To assess the effect of immunocastration on growth, we monitored body weight every two weeks for 40 weeks. No significant differences in final body weight were observed among the three groups at slaughter (IM vs. IC: *p* > 0.05; SC vs. IC: *p* > 0.05; IM vs. SC: *p* > 0.05) (Figure 3A), and their weekly growth trajectories were similar (Figure 3B), consistent with the characteristic slow growth of this breed. Although the dressing percentage was slightly lower in the IM group, no statistically significant differences were found among the groups (IM vs. IC: *p* > 0.05; SC vs. IC: *p* > 0.05; IM vs. SC: *p* > 0.05) (Figure 3C). Analysis of visceral organ weights revealed that the SC group had a significantly heavier lung than the IC group (IC vs. SC: *p* < 0.05) (Figure 3D), suggesting enhanced lipid deposition, a known consequence of surgical castration. No significant differences were detected in the weights of other organs (*p* > 0.05 for all pairwise comparisons). These data indicate that while immunocastration may slightly influence muscling, reflected in the lowest dressing percentage, the effect was not statistically significant. The highest dressing percentage in the SC group is likely attributable to combined hormonal suppression and increased fattening.

### 3.3. Effects on Testicular Size and Morphology

**Figure 4:**
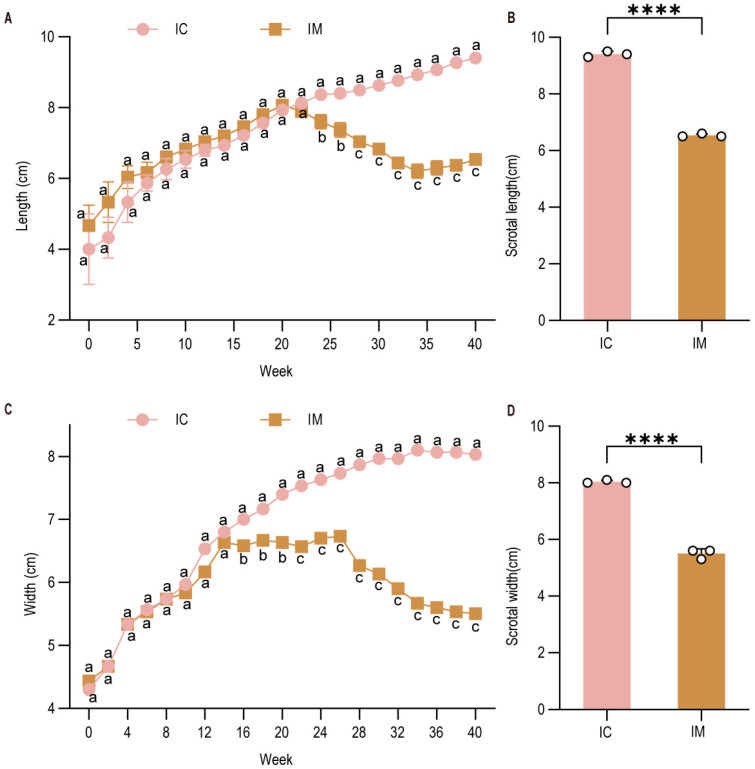
Testicular size was monitored through biweekly measurements of testicular dimensions (length and width), with developmental trajectories plotted over the experimental period. (**A**) Longitudinal changes in scrotal length across weeks; (**B**) Scrotal length; (**C**) Longitudinal changes in scrotal width across weeks; (**D**) Scrotal width. Data are presented as means ± SD. Different lowercase letters (a, b, c) indicate significant differences among groups at each time point: groups sharing the same letter are not significantly different (*p* > 0.05); groups with different adjacent letters (e.g., a vs. b) indicate *p* < 0.05; groups with non-adjacent letters (e.g., a vs. c) indicate *p* < 0.01. IC: Intact control group; IM: Immunocastration group.**** indicates a significant difference compared to the IC group at *p* < 0.0001.

**Figure 5:**
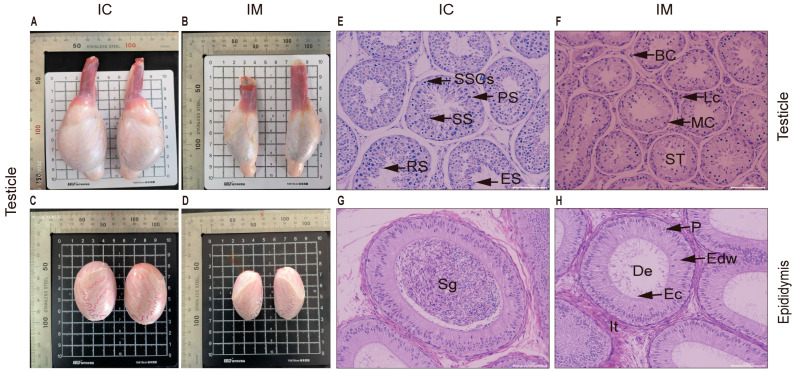
Effects of GnRH Immunization on Testicular Size and Histological Characteristics in Leizhou Goats. (**A**) Representative photograph of a testis from the IC group goat. Shown are the normal oval contour, well-developed body, attached corpus epididymis, and cauda epididymis. (**B**) Representative photograph of a testis from the IM group goat. Note the markedly reduced size and irregular shape of the testis, along with its loose attachment to the epididymis. (**C**) Testis from the IC group, demonstrating a well-developed morphology. (**D**) Representative testis from the IM group, showing volumetric atrophy. (**E**) Testicular histology of the IC group (200× magnification) demonstrating normal seminiferous tubules. (**F**) Photomicrograph of testicular tissue from the IM group at 200× magnification reveals vacuolization in the seminiferous tubules (ST) and a reduction in spermatozoa. (**G**) Photomicrograph of the epididymis from the IC group at 200× magnification shows well-defined stereocilia (Ec) lining the ductus epididymidis (De) lumen, which is packed with spermatozoa. (**H**) Photomicrograph of the epididymis from the IM group at 200× magnification shows a narrowed lumen of the ductus epididymidis (De) with a significant reduction in spermatozoa, which are scarcely observable. **Abbreviations**: ST = Seminiferous Tubule; BC = Blood Capillary; MC = Myoid cells; SSCs = Spermatogonial stem cells; PS = Primary Spermatocytes; SS = Secondary spermatocyte; RS = Round spermatids; ES = Elongating/Elongated spermatids; Lc = Interstitial cells; De = Ductus epididymidis; Sg = Sperm group; It = Interstitial tissue of epididymis; Edw = Epididymal duct wall; Ec = Epididymal cilia; P = Pseudostratified columnar epithelial cell nucleus. The non-English labels in the figure have no bearing on the scientific interpretation or presentation of the data.

Testicular development was monitored via biweekly measurements of scrotal length and width, respectively. The scrotal length in the IM group showed suppressed growth post-immunization, characterized by an initial slow increase followed by a decline, whereas the IC group exhibited rapid initial growth followed by a plateau (Figure 4A). Consequently, at the final measurement before slaughter at 40 weeks, the scrotal length in the IC group was significantly higher than that in the IM group (IM vs. IC: *p* < 0.0001) (Figure 4B). A similar trend was observed for scrotal width (Figure 4C,D). For precise post-mortem assessment, the testes and epididymides were collected and dissected free of surrounding tissues. Gross morphological examination revealed a pronounced reduction in the size of these organs in the IM group compared to the IC group (Figure 5A,B). Measurements confirmed that both the length and width of testes from the IC group were approximately 1 cm greater than those from the IM group (IC vs. IM: *p* < 0.01 for both length and width) (Figure 5C,D). Histological analysis of H&E-stained sections further corroborated these findings. The seminiferous tubules in the IM group were markedly atrophied, with a significant reduction in the area of both the tubules and the interstitial space compared to the IC group (IM vs. IC: *p* < 0.001 for tubular area; *p* < 0.01 for interstitial area) (Figure 5E,F). The tubules in the IM group displayed a shrunken lumen, a substantial decrease in the number of spermatogenic cells (including spermatogonia, primary and secondary spermatocytes, and round/elongated spermatids), and prominent vacuolization. In the epididymis, the IM group showed a drastically lower sperm density in the lumen (Figure 5H) compared to the dense sperm mass observed in the IC group (Figure 5G). The epididymal ducts in the IM group were also reduced in cross-sectional area (IM vs. IC: *p* < 0.01), with displaced epithelial cell nuclei and sparse stereocilia. Collectively, these data demonstrate a profound inhibitory effect of the vaccine on testicular development and spermatogenesis.

### 3.4. Assessment of Semen Quality

**Figure 6:**
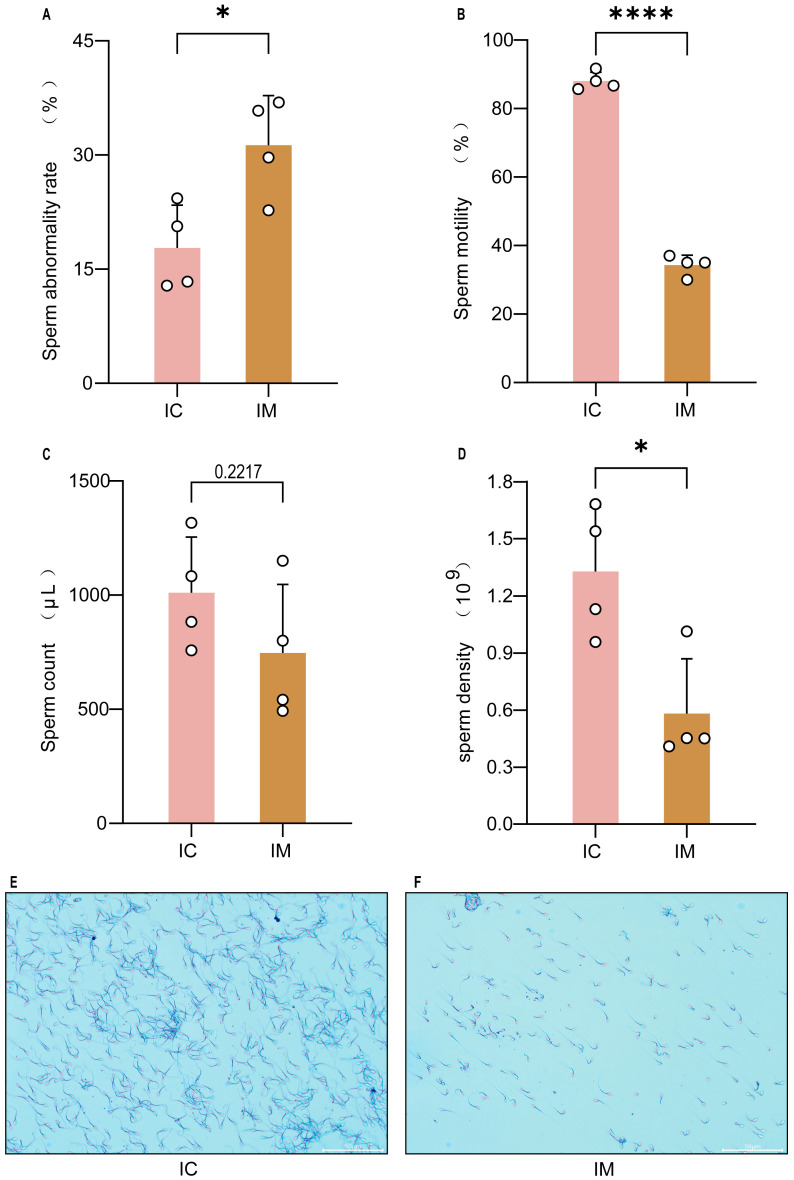
Effect of GnRH vaccine immunization on the sperm quality of Leizhou goats. (**A**) Comparison of sperm abnormality rate between groups. The total abnormality rate was significantly higher in the IM group than in the IC group (*p* < 0.05). (**B**) Comparison of sperm motility between groups. The total sperm motility was significantly reduced in the IM group (*p* < 0.01). (**C**) Comparison of semen volume between groups. The mean ejaculate volume in the IM group was numerically lower than that in the IC group, but the difference was not statistically significant. (**D**) Comparison of sperm concentration between groups. Sperm concentration was significantly decreased in the IM group (*p* < 0.05). (**E**) Sperm morphology in the IC group (HE staining, 200×). Shows a high-density population of morphologically normal spermatozoa with intact structure. (**F**) Sperm morphology in the IM group (HE staining, 200×). Characterized by a marked reduction in sperm number and a high incidence of abnormalities, including coiled tails and decapitated heads. Scale bar = 50 µm. * indicates a significant difference compared to the IC group at *p* < 0.05. **** indicates a significant difference compared to the IC group at *p* < 0.0001.

Semen was collected from bucks via electro-ejaculation, followed by comprehensive analysis of sperm quality. Compared to the IC group, the IM group exhibited a significant reduction in sperm density (*p* < 0.05, Figure 6A), total semen volume (*p* < 0.01, Figure 6B), and sperm motility (*p* < 0.001, Figure 6C). Furthermore, the percentage of morphologically abnormal sperm was significantly higher in the IM group (*p* < 0.05, Figure 6D), with a notable increase in the proportion of head and tail defects, predominantly manifested as bent and broken tails (Figure 6E,F). These results indicate that vaccination severely compromises sperm production, maturation, and overall semen quality.

### 3.5. Evaluation of Meat Quality

Meat quality, particularly in the context of immunocastration, was assessed through a comprehensive analysis of physical, chemical, and structural properties. The primary visual and sensory cues—color and pH—were measured to evaluate freshness and basic biochemical status. At both 45 min and 24 h post-slaughter, no significant differences were observed in lightness, redness, or yellowness across the IC, IM, and SC groups in the soleus, longissimus dorsi, or gastrocnemius muscles (Table 1). Similarly, pH values did not differ significantly among groups in any of the examined muscles (Table 2), indicating that neither castration method adversely affected these fundamental quality attributes.

**Figure 7:**
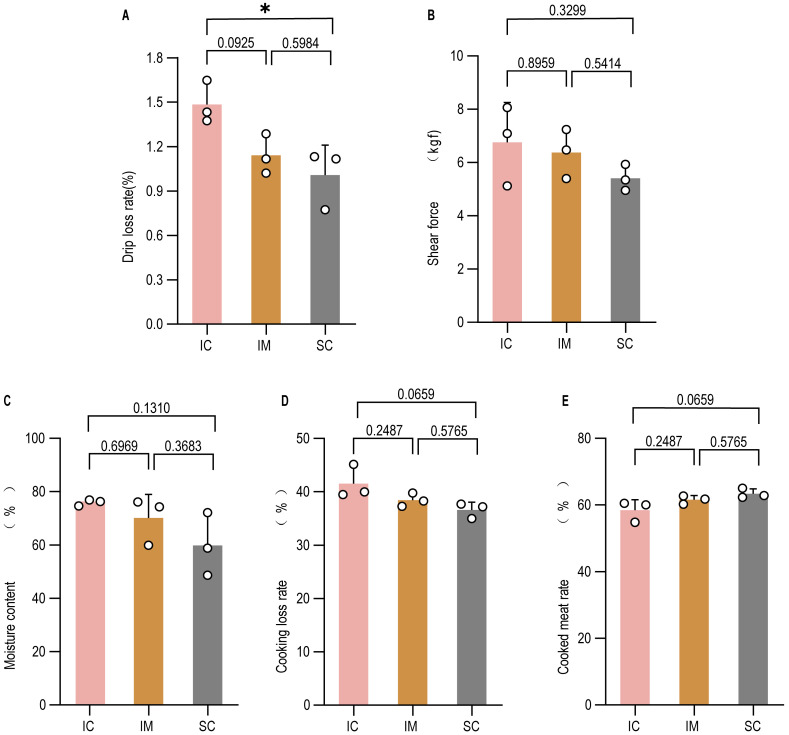
Meat quality indicators of Leizhou goats under three different treatment groups. (**A**) drip loss (%); (**B**) Warner–Bratzler shear force (N); (**C**) moisture content (%); (**D**) cooking loss (%); (**E**) cooking yield (%). GnRH immunization significantly reduced the drip loss percentage in goat meat ((**A**), *p* < 0.05), whereas no significant effects were observed on shear force (**B**), moisture content (**C**), cooking loss (**D**), or cooking yield (**E**) (*p* > 0.05). Data are presented as mean ± SEM. * *p* < 0.05.

As improving meat palatability is a primary objective of castration, we next evaluated key technological and sensory traits. The longissimus dorsi muscle from both the IM and SC groups exhibited numerically lower drip loss and shear force than the IC group (Figure 7A,B), with several comparisons reaching statistical significance. Moisture content was slightly reduced in the castrated groups, though not significantly different from the IC group (Figure 7C). Cooking loss and cooking yield showed no significant inter-group differences (Figure 7D,E). The consistent directional trends in drip loss and shear force between the IM and SC groups demonstrate that immunocastration can effectively enhance meat tenderness and water-holding capacity to an extent comparable to surgical castration.

**Figure 8:**
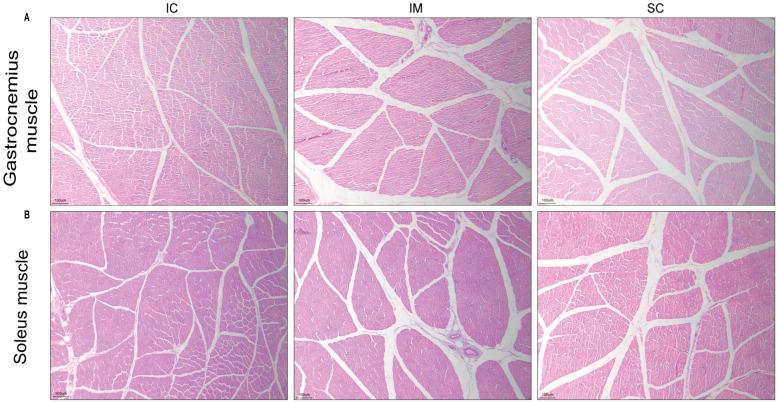
Histopathological evaluation of goat gastrocnemius and soleus muscles after GnRH immunization (H&E staining). (**A**) Representative transverse sections of gastrocnemius muscle from the three experimental groups; (**B**) Representative transverse sections of soleus muscle from the three experimental groups. Images display myofiber morphology, nuclear positioning, and connective tissue integrity. Original magnification: 100×. Scale bar: 100 μm.

To investigate the underlying structural basis for these improvements, histological analysis was conducted on the gastrocnemius and soleus muscles. Both the IM and SC groups showed a significant increase in intermuscular fat deposition (“marbling streaks”) compared to the IC group, alongside a trend toward reduced muscle fiber cross-sectional area (Figure 8A,B). The fat distribution patterns were remarkably similar between the two castrated groups. No significant differences in intramuscular fat content were detected. Critically, the muscle microstructure in the IM group remained intact and was histologically comparable to the SC group, featuring well-defined polygonal muscle fibers and preserved perimysial and endomysial connective tissue architecture.

## 4. Discussion

Immunocastration exerts its biological effect through functional suppression of the hypothalamic–pituitary–gonadal (HPG) axis rather than physical removal of gonadal tissue [20,35]. GnRH is secreted in a pulsatile manner from the hypothalamus and binds to GnRH receptors on anterior pituitary gonadotrophs, stimulating luteinizing hormone (LH) and follicle-stimulating hormone (FSH) release [36,37]. These gonadotropins drive Leydig cell steroidogenesis and Sertoli cell-mediated spermatogenesis [38]. Active immunization against GnRH induces circulating antibodies that bind endogenous GnRH, preventing receptor activation and thereby suppressing downstream testosterone production [20]. In contrast to surgical castration, which abruptly eliminates testicular steroidogenesis through orchiectomy, immunocastration produces a gradual endocrine attenuation determined by antibody kinetics and immune persistence [12,22].

In the present study, the GnRH(30) vaccine elicited sustained anti-GnRH antibody production in Leizhou goats, accompanied by marked testosterone suppression and severe impairment of spermatogenesis. Histological evidence of seminiferous tubule atrophy, germ cell depletion, vacuolization, and reduced epididymal sperm density directly reflects androgen deprivation. These histological changes are consistent with findings reported by Mustafa et al. [8] in rams and Paixão et al. [39] in boars, where GnRH-I immunization significantly suppressed spermatogenesis, characterized by complete absence of sperm within the seminiferous tubules. Similarly, Han et al. [20] demonstrated in rats that anti-GnRH immunization induced testicular atrophy and spermatogenic arrest. Testosterone is indispensable for maintaining seminiferous epithelium integrity and epididymal epithelial function [40]; therefore, the observed structural degeneration is physiologically consistent with functional hypogonadism induced by GnRH neutralization [41,42]. The morphological alterations in epididymal epithelium, including nuclear displacement within pseudostratified columnar cells, likely represent secondary consequences of androgen withdrawal, as epididymal differentiation and secretory activity are strongly androgen-dependent. These findings collectively confirm that the reproductive suppression observed in the IM group was mediated through endocrine axis inhibition rather than local tissue toxicity.

Our previous rodent studies demonstrated that the tandem-repeat GnRH(30) antigen could induce high-titer antibodies and suppress fertility for more than 36 weeks [23,24]. Because the mature GnRH decapeptide is highly conserved among mammals, cross-species immunogenicity was anticipated [43]. Based on interspecies body weight comparison and prior dose–response observations in rodents [23,24], the theoretical immunization dose for goats was determined to be 1.5 mg per animal in this study. Although the magnitude of testicular reduction in goats was less dramatic than the >90% reduction observed in rodents [23], the vaccine effectively suppressed testosterone and spermatogenesis, indicating adequate antigenicity in this larger ruminant species. The slightly attenuated response compared with rodents may reflect species-specific immune kinetics, differences in endocrine feedback regulation, or variability in antibody affinity maturation—highlighting the importance of considering species-specific factors in cross-species vaccine development, as previously emphasized in comparative immunological studies [44,45].

A key practical outcome was that immunocastration did not significantly impair early growth trajectories. Similar findings have been reported in model animals and other large livestock such as pig, cattle, and sheep [7,8,23,24,46,47]. However, some studies present contrasting perspectives [48,49,50]. Testosterone has anabolic effects mediated through androgen receptor signaling in muscle and bone; early suppression, such as that caused by pre-pubertal banding, may limit growth potential [2]. In this study, vaccination was administered during later growth stages, allowing early anabolic androgen exposure while subsequently reducing androgen-driven undesirable effects. This temporal endocrine modulation likely explains the absence of early growth retardation. In contrast, surgical castration induces an immediate and permanent cessation of androgen secretion, potentially altering metabolic partitioning more abruptly. The progressive endocrine decline induced by immunocastration may therefore represent a physiologically smoother transition.

Regarding carcass and meat physicochemical traits, both IM and SC groups demonstrated improved water-holding capacity and reduced shear force compared with intact controls [51,52,53]. These improvements are consistent with the known metabolic consequences of androgen withdrawal [54,55]. Testosterone promotes lean tissue accretion and limits adipogenesis [56]; its reduction shifts nutrient partitioning toward increased intramuscular fat deposition. Although intramuscular fat differences in our study were not statistically significant, histological observations and numerical trends support increased lipid deposition in castrated groups, which aligns with observations in bovine [57] and porcine models [58]. Enhanced lipid deposition improves perceived tenderness and juiciness [59], while altered muscle fiber characteristics and reduced collagen cross-linking may further contribute to decreased shear force [60]. The convergence of IM values toward those of surgical castration indicates that endocrine suppression achieved by the GnRH(30) vaccine functionally approximates orchiectomy with respect to muscle metabolic remodeling.

Water-related traits—including drip loss, cooking loss, and cooked meat yield—are closely associated with muscle structural integrity and postmortem protein–water interactions [61]. Androgen deprivation may influence calpain system activity [62], muscle fiber type composition [63], and intracellular water distribution [64]. The consistent directional changes across these parameters in both castration methods suggest that endocrine suppression, rather than the surgical procedure itself, is the primary determinant of meat quality improvement [65,66]. Thus, the comparable physicochemical profiles between IM and SC groups reinforce the conclusion that immunocastration reproduces the metabolic and structural consequences of surgical castration through endocrine modulation.

This study has limitations that warrant future investigation. Firstly, the sample size was modest and the observation period covered only part of the post-vaccination interval. Larger-scale trials spanning complete production cycles are therefore required to rigorously assess vaccine stability, economic return, and long-term health outcomes under commercial conditions. Secondly, although we documented previously unreported structural alterations, including epididymal deformities, the molecular and cellular mechanisms underlying these changes remain undefined. Future studies should establish quantitative relationships between antibody titers and physiological endpoints—such as the degree of testosterone suppression and the extent of tissue remodeling—and clarify the sources of inter-individual variation in immune responsiveness. Finally, tour analyses were largely restricted to growth performance and basic composition. A comprehensive assessment of eating quality will require measurement of key odor-related metabolites (e.g., androstenone and skatole), detailed fatty acid profiling, and systematic sensory evaluation to determine the broader impact of this approach on overall meat quality.

Collectively, evaluation of immune response, endocrine suppression, histomorphological alterations, semen quality decline, and carcass and meat physicochemical changes demonstrates that the GnRH(30) vaccine effectively induces functional hypogonadism in Leizhou goats. The treatment achieved reproductive suppression and meat quality enhancement comparable to surgical castration, while avoiding physical removal of gonadal tissue and without detectable adverse effects on early growth. These findings indicate that the observed phenotypic changes across reproductive, growth, and meat quality variables can be directly attributed to controlled suppression of the HPG axis.

## 5. Conclusions

Immunization with the tandem-repeat GnRH(30) vaccine produced a sustained humoral response that effectively suppressed circulating testosterone and impaired reproductive function in male Leizhou goats. The magnitude of endocrine suppression and testicular regression was comparable to that observed following surgical castration, indicating that active immunization achieved functional reproductive inhibition equivalent to physical gonad removal. Importantly, growth performance was not adversely affected, suggesting that reproductive suppression did not compromise somatic development under the present management conditions. In parallel, both immunocastrated and surgically castrated goats exhibited improvements in meat quality traits, including reduced shear force and drip loss, accompanied by increased fat deposition. Collectively, the treatment effects across endocrine, reproductive, growth, and carcass-related variables demonstrate that GnRH immunocastration provides effective reproductive control while maintaining productive performance and improving meat quality in this indigenous goat breed.

## Figures and Tables

**Table 1:** Meat color of soleus muscle, longest dorsal muscle, and gastrocnemius muscle in three groups.

Parameter		IC		IM		SC	
		45 min	24 h	45 min	24 h	45 min	24 h
Soleus muscle	L*	52.00 ± 2.91	48.70 ± 1.37	53.07 ± 0.42	49.23 ± 1.33	54.07 ± 1.33	50.77 ± 0.49
Longest dorsal muscle		53.97 ± 2.96	48.27 ± 5.05	54.40 ± 4.89	49.77 ± 1.67	55.27 ± 0.55	51.43 ± 2.01
Gastrocnemius muscle		46.93 ± 3.13	44.53 ± 2.18	46.67 ± 5.21	43.77 ± 4.40	51.67 ± 4.13	49.47 ± 2.48
Soleus muscle	a*	14.33 ± 0.40	14.97 ± 0.97	13.33 ± 1.46	17.07 ± 0.51	14.8 ± 1.15	15.4 ± 0.46
Longest dorsal muscle		11.97 ± 1.11	13.50 ± 0.87	12.20 ± 1.13	15.5 ± 2.72	13.73 ± 0.60	15.97 ± 2.65
Gastrocnemius muscle		14.53 ± 0.95	13.30 ± 0.72	13.23 ± 1.42	13.07 ± 2.50	14.43 ± 2.33	16.33 ± 2.12
Soleus muscle	b*	4.97 ± 1.27	8.63 ± 0.67	4.27 ± 0.95	8.77 ± 0.75	4.50 ± 0.72	7.87 ± 0.21
Longest dorsal muscle		2.93 ± 0.74	8.00 ± 1.56	4.30 ± 1.99	8.70 ± 1.25	3.97 ± 0.38	8.63 ± 1.60
Gastrocnemius muscle		6.63 ± 1.78	8.40 ± 0.95	6.97 ± 1.64	9.30 ± 1.25	5.20 ± 1.66	8.33 ± 1.62

Note: L* (lightness) indicates the degree of brightness. a* (redness) represents the red intensity. b* (yellowness) corresponds to the yellow intensity. In goat meat quality evaluation, these values in the table were collected at the time points of 45 min and 24 h post-slaughter, respectively. A lower L* and b* value, coupled with a higher a* value, are generally associated with a more desirable meat color.

**Table 2:** pH value in soleus muscle, longest dorsal muscle, and gastrocnemius muscle of three groups.

Parameter	IC		IM		SC	
	45 min	24 h	45 min	24 h	45 min	24 h
Soleus muscle	6.47 ± 0.21	5.57 ± 0.05	6.48 ± 0.25	5.51 ± 0.06	6.45 ± 0.57	5.81 ± 0.17
Longest dorsal muscle	6.61 ± 0.26	5.71 ± 0.26	6.14 ± 0.41	5.71 ± 0.41	6.43 ± 0.26	5.39 ± 0.07
Gastrocnemius muscle	6.33 ± 0.19	5.44 ± 0.11	5.81 ± 0.32	5.61 ± 0.14	6.39 ± 0.29	5.54 ± 0.14

Note: All pH values in the table were collected at the time points of 45 min and 24 h post-slaughter, respectively.

## Data Availability

The original contributions presented in this study are included in the article. Further inquiries can be directed to the corresponding authors.
